# In Vitro Evaluation of Photodynamic Activity of Plant Extracts from *Senna* Species against Microorganisms of Medical and Dental Interest

**DOI:** 10.3390/pharmaceutics15010181

**Published:** 2023-01-04

**Authors:** Analú Barros de Oliveira, Túlio Morandin Ferrisse, Sarah Raquel de Annunzio, Maria Gleiziane Araújo Franca, Maria Goretti de Vasconcelos Silva, Alberto José Cavalheiro, Carla Raquel Fontana, Fernanda Lourenção Brighenti

**Affiliations:** 1 Department of Orthodontics and Pediatric Dentistry, School of Dentistry, São Paulo State University (UNESP), Araraquara 14801-903, SP, Brazil; 2 Department of Dental Materials and Prosthodontics, School of Dentistry, São Paulo State University (UNESP), Araraquara 14801-903, SP, Brazil; 3 Department of Clinical Analysis, School of Pharmaceutical Sciences, São Paulo State University (UNESP), Araraquara 14801-903, SP, Brazil; 4 Department of Organic and Inorganic Chemistry, Federal University of Ceará, Fortaleza 60020-181, CE, Brazil; 5 Department of Biochemstry and Organic Chemistry, Chemistry Institute, São Paulo State University (UNESP), Araraquara 14800-060, SP, Brazil

**Keywords:** antimicrobial photodynamic therapy, natural photosensitizer, *Senna* spp., bioprospecting

## Abstract

Background: Bacterial resistance requires new treatments for infections. In this context, antimicrobial photodynamic therapy (aPDT) is an effective and promising option. Objectives: Three plant extracts (*Senna splendida*, *Senna alata*, and *Senna macranthera*) were evaluated as photosensitizers for aPDT. Methods: *Cutibacterium acnes* (ATCC 6919), *Streptococcus mutans* (ATCC 35668), *Staphylococcus aureus* (ATCC 25923), *Escherichia coli* (ATCC 25922), and *Candida albicans* (ATCC 90028) were evaluated. Reactive oxygen species production was also verified. Oral keratinocytes assessed cytotoxicity. LC-DAD-MS analysis identified the chemical components of the evaluated extracts. Results: Most species cultured in the planktonic phase showed total microbial reduction (>6 log10 CFU/mL/*p* < 0.0001) for all extracts. *C. albicans* cultured in biofilm showed total microbial reduction (7.68 log10 CFU/mL/*p* < 0.0001) for aPDT mediated by all extracts. Extracts from *S. macranthera* and *S. alata* produced the highest number of reactive oxygen species (*p* < 0.0001). The *S. alata* extract had the highest cell viability. The LC-DAD-MS analysis of active extracts showed one naphthopyrone and seven anthraquinones as potential candidates for photoactive compounds. Conclusion: This study showed that aPDT mediated by *Senna* spp. was efficient in microbial suspension and biofilm of microorganisms of medical and dental interest.

## 1. Introduction

Bacterial resistance develops at a higher rate than the industry can produce new drugs, increasing morbidity from infections easily treated in the past [1]. Recent studies indicate that increased bacterial resistance could cause over 300 million deaths worldwide in the future [2]. Therefore, developing unconventional treatments against these microorganisms is highly relevant.

The *Streptococcus*, *Staphylococcus*, *Escherichia*, *Corynebacterium*, and *Candida* genera are among the main etiological agents of medical and dental diseases. These microorganisms can cause lesions in the skin, oral cavity, and vaginal mucosa. They are also associated with invasive infections in hospital patients, dental caries, aggressive periodontitis, endodontic lesions, gastro-urinary infections, sepsis, and endocarditis [3,4,5,6].

Photodynamic therapy (PDT) combines photophysical and photochemical mechanisms that generate a biological response using specific light sources and an oxygen-associated photosensitizing agent. Furthermore, PDT does not induce microbial resistance [7,8]. Classically, there are two photochemical mechanisms involved in PDT, called Type I and Type II. When the photosensitizer (PS) absorbs photon light, the electron in the highest occupied molecular orbital (HOMO) state is excited into the lowest excited molecular orbital (LUMO) state, producing the first excited singlet state (^1^PS) [9]. Next, a fraction of ^1^PS can undergo an intersystem crossing process originating an electron in excited triplet state (^3^PS) [9]. Then, ^3^PS can also undergo an electron transfer reaction (Type I mechanism) in the presence of an electronic donor to produce a PS radical anion. The PS radical anion establishment is crucial, since its presence can originate superoxide radical anion (O_2_^−^), hydrogen peroxide (H_2_O_2)_, and hydroxyl radical (_1_OH^−^). Dioxygen (^3^O_2_) is a molecule in an electronic triplet state which can be found in natural ground. This molecule undergoes a physical energy transfer process called Type II, which produces the ground state singlet oxygen molecule ^1^O_2_ [9]. The O_2_^−^, H_2_O_2, 1_OH^−^, and ^1^O_2_ molecules are responsible for the biological response after PDT application [9].

There is a wide range of natural photosensitizers in PDT [10]. The advantages of natural compounds are their adherence to the cytoplasmic membrane and the potential to produce reactive oxygen species [11]. Some photosensitizers have limitations, such as slow excretion after use, prolonged patient sensitization, the formation of aggregates that hinder photosensitizers from absorbing light, low water solubility, and different therapeutic windows. Thus, plant materials might be an appealing source for discovering new photosensitizers that could be safer and more efficient [12]. Therefore, the present study aimed to evaluate the in vitro potential of *Senna* spp. extracts as photosensitizers for antimicrobial photodynamic therapy (aPDT) against microorganisms of dental and medical interest cultured in suspension and biofilm. It also investigated the cytotoxic effects of *Senna* spp. extracts in the presence and absence of light on human keratinocyte cells.

## 2. Materials and Methods

### 2.1. Preparation of Senna spp. Extracts

Three plant extracts (*Senna macranthera* I&B, *Senna splendida* I&B, and *Senna alata* L.) were obtained from the native flora of northeastern Brazil. Supplementary Table S1 describes the plant materials of the present study.

The plants were registered in the National System of Genetic Resource Management and Associated Traditional Knowledge (SisGen; Register #AB06D11), and all collections were made with the permission of the Brazilian Institute of Environment and Renewable Natural Resources (IBAMA) through the System of Authorization and Information on Biodiversity (SISBIO), which provided proof of registration (SISBIO; Register #55774).

The raw materials to produce *Senna macranthera*, *Senna splendida*, and *Senna alata* extracts were leaves, leaves, and branches, respectively. Initially, the plant materials were dehydrated at 40 °C for 36 h, crushed, and extracted in an ultrasonic bath at a ratio of 100 mg of powder to 3 mL of methanol. Next, the extracts were filtered and concentrated in a vacuum in a rotary evaporator [13]. The mass yield of the extracts was determined with the equation: (yield = [final mass/initial mass] × 100).

For biological tests, the plant extracts were diluted in dimethyl sulfoxide (DMSO; Labsynth, Brazil) (5 mg/mL) and then in culture medium for a final concentration of 0.05 mg/mL or 0.50 mg/mL. All plant extract solutions were freshly prepared in a light-protected environment.

### 2.2. Chemical Characterization of the Crude Extracts

The extracts were analyzed on an LC-DAD-MS Shimadzu/Bruker system (Kyoto, Japan) configurated with an LC-20AD quaternary pump, a CTO-20A column oven, a SIL-20AHT autosampler, an SPD-M20A diode array detector, a CBM-20A communication unit, and an ion trap mass spectrometer (Amazon SL, Bruker, Billerica, MA, USA) with electrospray ionization (ESI). The spectrometer analysis parameters were 7.0 kV capillary, ESI in negative and positive ion modes, 500 V end plate offset, 50.0 psi nebulizer, dry gas (N2) flow rate of 10.0 L/h, and temperature of 300 °C. Spectra (*m*/*z* 50–1000) were recorded every 2.0 s. Separation occurred with a C18 analytical column (Phenomenex Luna™ 5 μm C18(2) 100 Å, 250 × 4.6 mm^2^). The chromatographic conditions were a flow rate of 1.0 mL/min, column temperature of 35 °C, and injection volume of 10 μL. The mobile phase consisted of water (A) and acetonitrile (B), both with 0.1% formic acid, with the following linear gradient elution: 2/30/40/45 min 5/45/100/100% of B, returning to the initial conditions of 2% B over 3 min, and holding it for a further 12 min for column reconditioning. Absorption spectral data were collected within 45 min from 200 to 800 nm, and chromatograms were registered at 450 nm. HRMS data were acquired on a UPLC-QTOF Waters™ that is a UPLC Acquity H-Class in line with a Xevo G2-XS QT of an MS spectrometer. The photoactive compounds were annotated based on data from UV/Vis spectra, MS/MS, HRMS, and the literature.

### 2.3. Light Sources

The visible light absorption spectrum was measured with a Synergy H1M microplate fluorescence reader (Synergy H1a Multi Mode Reader, Biotek, Winooski, VT, USA). The light sources consisted of LEDs (IrradLED™—biopdi, São Carlos, SP, Brazil) without optical fiber. All plant extracts were light-activated at 450 nm (151 mW/cm^2^).

### 2.4. Bacterial Strains and Culture Conditions

The strains used in this study were *Cutibacterium acnes* (ATCC™ 6919™), *Candida albicans* (ATCC™ 90.028™), *Escherichia coli* (ATCC™ 25.922™), *Staphylococcus aureus* (ATCC™ 25.923™), and *Streptococcus mutans* (ATCC™ 35.668™) from the National Institute for Quality Control in Health (INCQS) of the Oswaldo Cruz Foundation (FIOCRUZ—Manguinhos, RJ, Brazil). Strain reactivation and microbial inoculum adjustments were performed according to the parameters described in Supplementary Table S2. The inoculum was adjusted with a spectrophotometer (Biotek™ ELx800—Winooski, VT, USA) reading at 630 nm.

### 2.5. Antimicrobial Photodynamic Therapy (aPDT) Using Microbial Suspensions

Microbial suspensions were prepared as described in item 2.4 with optical density assessments (λ = 630 nm). A volume of 50 μL of the prepared inoculum was transferred to each well plate containing 50 μL of plant materials, resulting in a volume of 100 μL per well and diluting solutions and the inoculum by 50%.

Six groups were studied: negative control, plant material, aPDT (light + plant material), and vehicle control without and with exposure to light. Plant material and aPDT groups were pre-incubated with plant materials for five minutes in the dark and at room temperature. Light and negative control groups were pre-incubated in a culture medium for five minutes in the dark and at room temperature. Irradiation was performed in the light and aPDT groups at 450 nm and 80 J/cm^2^ (Table 1). Irradiation time was 18 min in fractional mode. Plant material and negative control groups were not irradiated and remained at room temperature for 18 min. The experiments were performed on two occasions (*n* = 10) and in duplicate.

After the treatment, the suspensions were diluted in a culture medium, and 5 μL of each dilution was plated with the agar drop method [14], as described in Supplementary Table S2. The number of cultured colonies was counted after 24 or 48 h of incubation under the conditions described in Supplementary Table S2.

### 2.6. Antimicrobial Photodynamic Therapy (aPDT) in Biofilm

The biofilm model was developed with an in vitro technique by Fontana et al. (2009) [15], using the same parameters described in item 2.5. After preparing the microbial suspension, each plate well was filled with 150 μL of the inoculum and incubated according to the parameters described in Supplementary Table S2 for three days. PDT was performed after 48 h when the biofilm reached a mature stage.

A volume of 50 μL of plant extracts (0.05 mg/mL or 0.50 mg/mL) was added to each well containing the biofilm. Biofilms were pre-incubated with the plant extracts for 15 min before aPDT. After the treatment, the biofilm was gently detached and diluted in a culture medium for further cultivation and colony counting (CFU/mL). Table 1 describes the used parameters and experimental groups.

### 2.7. Reactive Oxygen Species (ROS) Detection—Cell-Free System (Solution)

The detection test of reactive oxygen species used fluorescent probes 3′-p-(aminophenyl) fluorescein (APF; detects mainly hydroxyl radical [•OH]) and Singlet Oxygen Sensor Green (SOSG; detects singlet oxygen [^1^O_2_]) (Invitrogen, Thermo Fischer Scientific, Waltham, MA, USA) and was handled according to the manufacturer’s instructions.

The solutions with plant materials were prepared in a phosphate buffer (0.1 M; pH of 7.2), added to the solutions of 3 μmol/mL of APF or SOSG in the wells of a black flat-bottom 96-well plate (Corning™, Nova York, EUA), and irradiated from above to archive the desired energy dose. Fluorescence readings were taken immediately after irradiation using the Synergy H1M (Synergy H1 Multi-Mode Reader, BioTek, Winooski, VT, USA). Excitation/emission wavelengths for APF were 490/515 nm, and for SOSG they were 505/525 nm.

### 2.8. Cytotoxicity Assessment

Cells from oral keratinocyte lines (NOK-SI) were cultured in 75-cm^2^ cell culture flasks and maintained in an incubator at 37 °C in 5% CO_2_ (MCO-17AC, Sanyo Electric Co., Ltd., Osaka, Japan). The DMEM culture medium (Dulbecco’s Modified Eagle’s Medium, Dulbecco’s Modified Eagle’s Medium, Campinas, SP, Brazil) supplemented with 10% fetal bovine serum (Cultilab, Campinas, SP, Brazil) was renewed every 48 h.

Triton X-100 was used as a negative control and DMEM supplemented with 10% FBS was used as growth control. The cells were trypsinized after reaching 80% confluence. The suspension containing 1.7 × 10^5^ cells/mL of each cell line was seeded in 96-well plates and incubated for 24 h in 5% CO_2_ [16]. The cells were treated with plant extracts (50 mg/mL) and controls for 15 min before incubation and then 30 min of irradiation, as described in item 2.6. Cell viability was assessed with the MTT assay [17] by adding 100 μL of MTT (3 mg/mL, Sigma Aldrich, San Luis, MO, EUA) to each plate. After three hours, absorbance was read at 562 nm, as indicated by the manufacturer (Datasheet Sigma Aldrich, catalog no. M2128). The experiments were performed in triplicate. For this step, it was used the same extract’s concentrations (mg/mL) and light doses (J/cm^2^) employed in microbial suspensions and biofilm evaluation.

### 2.9. Statistical Analysis

The data from pilot studies were used to calculate the sample size for the following parameters: the minimum difference between means (0.0186), standard deviation (0.0243), the number of repetitions (3), power of analysis = 0.80, and α = 0.05. The IBM SPSS 20.0 program analyzed the data. The Shapiro–Wilk and Levene’s tests assessed normal distribution and homoscedasticity, respectively. A two-way analysis of variance was applied to each microorganism, ROS detection and for cytotoxicity assay. Comparative analysis was performed by estimating means with a 95% confidence interval. A correlation study between the probe in ROS detection and the natural products with the highest CFU reduction (log-reduction > 3.0) was performed with Pearson’s correlation coefficient. The statistician was blind.

## 3. Results

### 3.1. Chemical Characterization of the Crude Extracts

**Figure 1:**
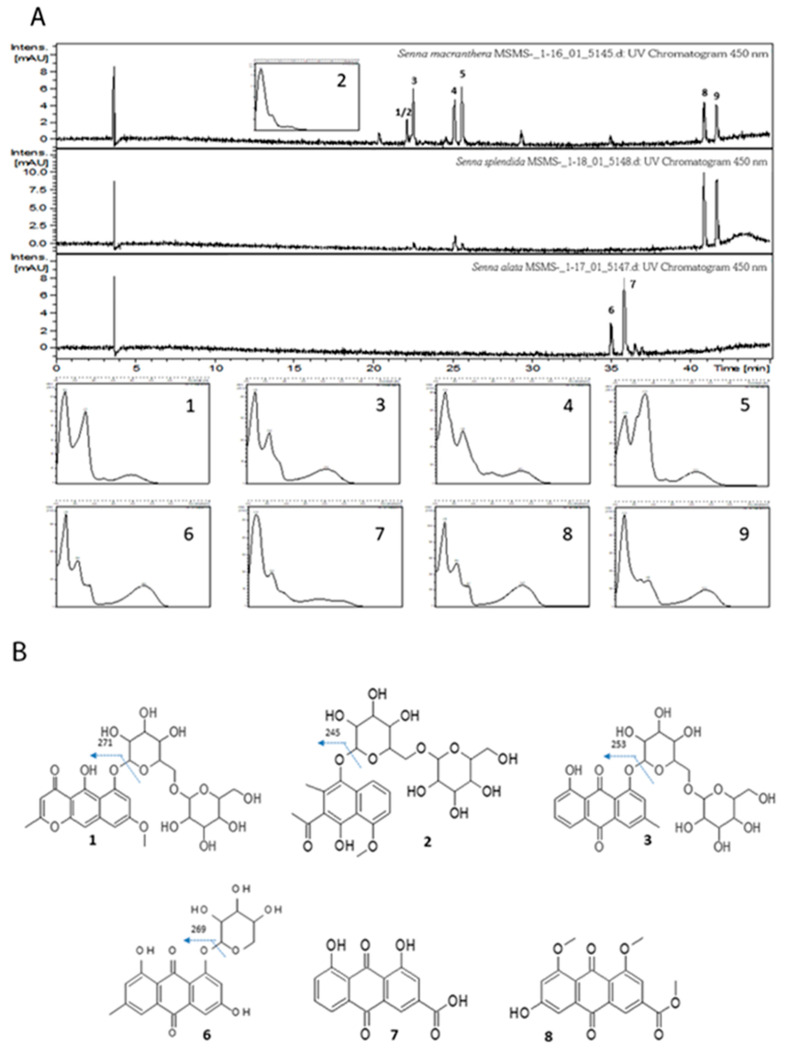
(**A**) Chromatograms of *Senna* extracts registered at 450 nm and UV/Vis spectra of photoactive compounds; (**B**) Molecular structure of some photoactive compounds.

The *Senna* extract chromatograms obtained by HPLC, with detection at 450 nm (Figure 1A), indicated a similar composition of photoactive compounds in leaves of *S. macranthera* and *S. splendida*, with four bands in each extract. *S. alata* showed two photoactive compounds in the twig extract. Considering taxonomic uncertainties involving species of the *Senna* and *Cassia* genera, substances in these plant genera, as reported in the literature, with absorption bands in the region of 400–450 nm, represented potential candidates when annotating the detected photoactive compounds. Therefore, anthraquinones, chromenes, benzochromenones (naphthopyrones), xanthones, and anthrones reported in species of these two genera were considered and compared with experimental data from UV/Vis, MS/MS, and HRMS [18]. The annotated compounds included one naphthopyrone (Figure 1(B1)), one acetonaphthone (Figure 1(B2)), four anthraquinones (Figure 1(B3,B6–B8)) (Table 2 and Figure 1), and three unidentified compounds (Figure 1(A4,A5,A9)) that could be anthraquinones based on their UV/Vis spectra (Figure 1A). The phytochemical literature does not show HRMS data compatible with the experimental data, making these compounds potentially unpublished. Studies aimed at the isolation and structural elucidation of these substances are in progress.

### 3.2. Visible Light Absorption Spectrum

The absorption spectra of the three plant extracts revealed a large absorption band covering the blue wavelength spectrum (400 nm), indicating a potential application of these three plant materials to aPDT (Supplementary Figure S1).

### 3.3. Antimicrobial Photodynamic Therapy (aPDT) Using Microbial Suspensions

There were significant differences in the interaction between natural substances and exposure to light (aPDT) (*p* < 0.0001) for all the studied microbial species (Table 3 and Table S3).

**Figure 2:**
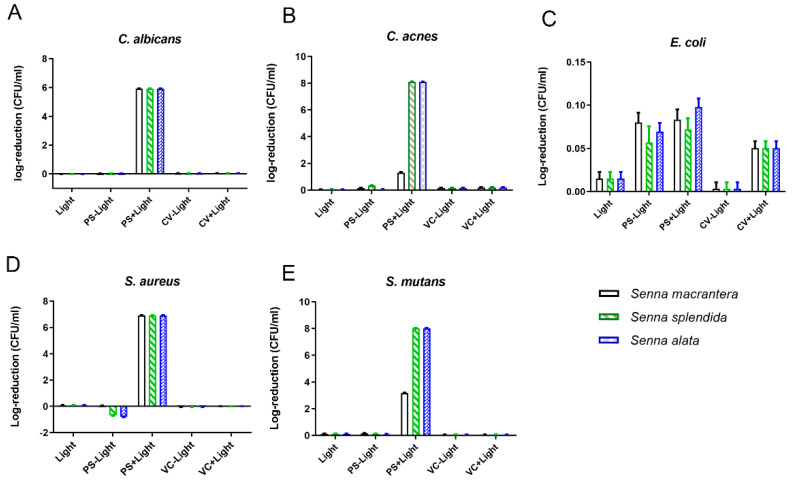
Effect of PDT of plant extracts (0.05 mg/mL) on microorganisms in suspensions (mean ± 95% confidence interval). (**A**) = *Candida albicans*; (**B**) = *Cutibacterium acnes*; (**C**) = *Escherichia coli*; (**D**) = *Staphylococcus aureus*; (**E**) = *Streptococcus mutans*. PS − light = plant material not exposed to light; PS + light = plant material exposed to light; VC − light = vehicle control not exposed to light; VC + light = vehicle control exposed to light.

For *C. albicans*, total reduction (5.93 log CFU/mL) occurred after PDT with *S. splendida*, *S. alata*, or *S. macranthera* (Figure 2A). *C. acnes* showed a 1.30 log CFU/mL reduction after PDT with *S. macranthera*. Total bacterial reduction (7.98 log CFU/mL) occurred after PDT with *S. splendida* and PDT with *S. alata* (Figure 2B). However, there was no significant reduction in *E. coli* (Figure 2C). Additionally, *S. aureus* showed reductions of 1.06 log CFU/mL and 2.24 log CFU/mL after PDT with *S. alata*. Total bacterial reduction (6.92 log CFU/mL) occurred after PDT with *S. macranthera* or *S. splendida* (Figure 2D). *S. mutans* showed a bacterial reduction of 3.19 log CFU/mL after PDT with *S. macranthera*. Total bacterial reduction (8.02 log CFU/mL) occurred after PDT with *S. splendida*, *S. macranthera*, or *S. alata* (Figure 2E).

### 3.4. Antimicrobial Photodynamic Therapy (aPDT) in Biofilm

There were statistically significant differences for the treatment with natural substances and exposure to light (*p* < 0.0001) and the interaction between natural substances and exposure to light (*p* < 0.0001) for *C. acnes* (extract concentration of 0.5 mg/mL), *C. albicans* (extract concentrations of 0.05 mg/mL and 0.5 mg/mL), *S. aureus* (extract concentration of 0.5 mg/mL), and *S. mutans* (extract concentrations of 0.05 mg/mL and 0.5 mg/mL) (Table 4 and Table S4). As observed in the suspensions, biofilm reduction depends on plant material light absorption.

**Figure 3:**
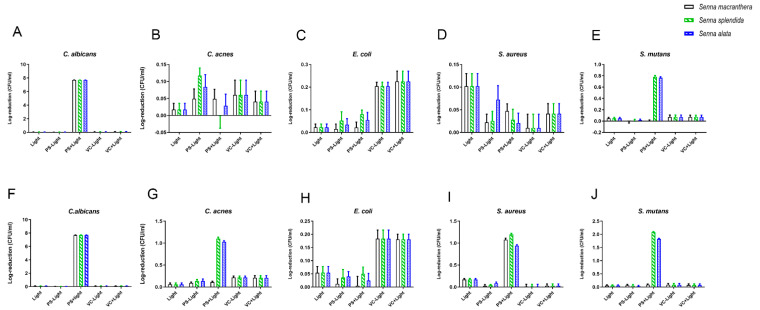
Effect of PDT of plant extracts ((**A**–**E**) = 0.05 mg/mL and (**F**–**J**) = 0.5 mg/mL) on microorganisms in biofilms (mean ± 95% confidence interval). (**A**,**F**) = Candida albicans. (**B**,**G**) = Cutibacterium acnes. (**C**,**H**) = Escherichia coli. (**D**,**I**) = Staphylococcus aureus. (**E**,**J**) = Streptococcus mutans. PS − light = plant material not exposed to light; PS + light = plant material exposed to light; VC light = vehicle control not exposed to light; VC + light = vehicle control exposed to light.

When irradiated, *S. macranthera* had the lowest antimicrobial effect on *C. acnes* (Figure 3B,G), *E. coli* (Figure 3C,H), and *S. mutans* (Figure 3E,J) at both tested concentrations (0.05 mg/mL and 0.5 mg/mL). The highest reduction for this plant extract occurred for *C. albicans* at both tested concentrations (Figure 3A,F). This extract promoted total microbial reduction (7.7 log CFU/mL) for *C. albicans*.

*S. splendida* at 0.05 mg/mL showed a significant reduction for *C. albicans* biofilm (Figure 3A) and total microbial reduction (7.7 log CFU/mL). At 0.5 mg/mL, reductions of 1.10 log CFU/mL, 1.19 log CFU/mL, and 2.08 log CFU/mL log occurred after PDT with *C. acnes* (Figure 3G), *S. aureus* (Figure 3I), and *S. mutans* (Figure 3J), respectively.

*S. alata* at 0.5 mg/mL promoted reductions of 0.94 log CFU/mL, 1.02 log CFU/mL, and 1.82 log CFU/mL after PDT with *S. aureus* (Figure 3I), *C. acnes* (Figure 3G), and *S. mutans* (Figure 3J), respectively. Total bacterial reduction in *C. albicans* (7.68 log CFU/mL) occurred after PDT at both tested concentrations (0.05 mg/mL and 0.5 mg/mL) (Figure 3A,F).

### 3.5. ROS Detection

**Figure 4:**
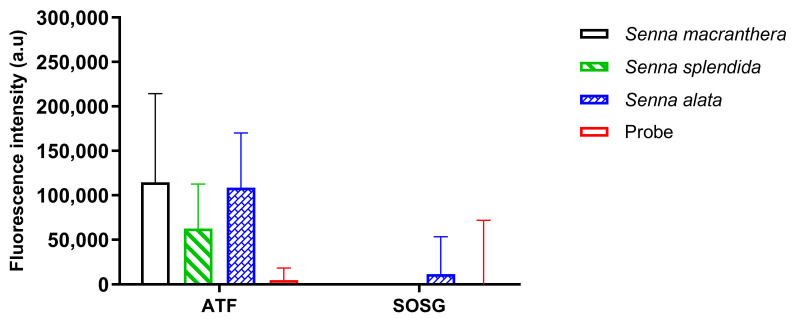
ROS production in plant extracts after exposure to light (mean ± 95% confidence interval).

ROS was detected in all plant extracts. There were significant results in the type of probe and plant material (*p* < 0.0001) (Supplementary Table S5). *Senna* spp. produced more _1_OH^−^ than O_2_ radicals. Additionally, only *S. alata* produced the O_2_ radical, but it was not statistically significant compared to the control (only probe). *S. macranthera* and *S. splendida* were the plant materials that produced the highest amount of the _1_OH radical, whereas *S. alata* also produced it but at a lower amount (Figure 4).

### 3.6. Cytotoxicity Assessment

**Figure 5:**
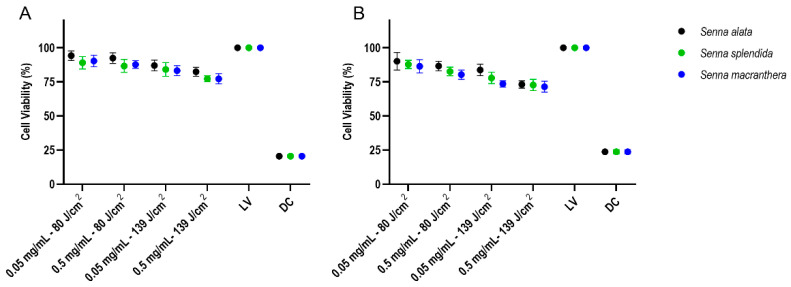
Percentage of cell viability of the oral keratinocyte culture after treatment with plant materials not exposed (**A**) and exposed (**B**) to light. Two-way ANOVA with graphics with mean ± confidence interval.

When oral keratinocytes were exposed to light the great significant difference was found between the live control and dead control. In addition, with the higher levels of extracts concentrations (mg/mL) and light dose (J/cm^2^) there was a reduction in cell viability (Figure 5A). A suitable reduction in oral keratinocytes was found when these cells were exposed to light. The reduction in viability was higher for all extracts with concentration of 0.5 mg/mL and irradiated with 139 J/cm^2^. However, even with higher extract’s concentrations and light doses, for all extracts there was a cell viability higher than 70% (Figure 5B). Details about the statistical analysis can be found in Table 5.

### 3.7. Correlation Study

The correlation study was conducted to evaluate whether log reduction can be directly associated with the _1_OH radical generation. These studies were performed between microbial species and plant extracts that reached a minimum of 3 log reduction. *S. splendida* showed a significant correlation to *C. albicans* (*p* = 0.0230/r = 0.8733/R^2^ = 0.7627) and *S. aureus* (*p* = 0.0003/r = 0.9863/R^2^ = 0.9727). There was also a significant correlation between *S. macranthera* and *S. aureus* (*p* = 0.0020/r = 0.9628/R^2^ = 0.9271). Lastly, *S. alata* showed a significant correlation for *C. acnes* (*p* = 0.0359/r = 0.8409/R^2^ = 0.7071) and *S. aureus* (*p* = 0.0020/r = 0.9636/R^2^ = 0.9285) (Supplementary Table S6).

## 4. Discussion

According to Miethke (2021) [19], the development of new antimicrobial substances is a complex, long, and expensive task. Thus, establishing alternatives for treating infections caused by resistant microorganisms is a topic of great interest. In this context, PDT has been studied as a therapeutic option for treating diseases caused by microorganisms, aiming to minimize the side effects of commonly used therapies [20].

The interest in phytomedicine has increased in recent years. Many plants have phytotherapeutic properties that may represent an alternative to classic photosensitizer compounds. However, the literature is sparse regarding the information on the quality, safety, and efficacy of natural products for antimicrobial photodynamic therapy [10].

The hypothesis tested in the present study was whether the crude extracts of *Senna* spp. could serve as photosensitizers in aPDT. The study identified the absorption spectra of the crude extracts of *S. splendida*, *S. alata*, and *S. macranthera* and selected the light source for irradiation according to each spectrum. To the best of our knowledge, this is the first study to evaluate the efficacy of PDT mediated by these plant extracts.

The crude extracts of *Senna* spp. showed absorption peaks within the spectral range of 400 to 500 nm. This absorption is pertinent for aPDT applications because it is within the spectral range known as the therapeutic window [21].

Furthermore, in the photoactivity experiments with microbial suspensions, all plant materials showed a microbial reduction higher than 3 log CFU/mL for at least one of the studied microorganisms, except for *E. coli*. Additionally, the literature has not reported similar results for suspensions of *C. acnes*, *C. albicans*, and *S. mutans*. Microbial biofilms showed a total reduction in *C. albicans* in all plant materials, and overall, *S. splendida* and *S. alata* promoted a total microbial reduction in more microorganisms.

Our results show that *Senna* spp. extracts are promising as photosensitizers. This reduction is among the factors that predict whether an antimicrobial compound will be clinically significant [20]. Moreover, it is worth noting that the plant material concentration is below the maximum recommended dose for plant material use [22].

There is no consensus in the literature about the optimal light dose for PDT [23]. The light dose must be adjusted to each studied microorganism and photosensitizer. The light dose used in the present study was based on a previous one [24]. The literature shows many scientific reports about using natural photosensitizers in antimicrobial photodynamic therapy (aPDT) [10,25].

Nonetheless, the motivation of the present study was that the reported plant materials have shown several limitations, such as low cell permeability; different absorption band from the desired therapeutic window; high permanence in some tissues, leaving the patient photosensitive for weeks; the formation of toxic secondary metabolites after irradiation; and limited antimicrobial capacity.

Although natural photosensitizing agents differ from this study, the light exposure time stands out. In the present study, the therapy was successful against the microorganisms in both suspension and biofilm, with much shorter irradiation times.

In this context, curcumin, Chlorella (green natural microalga) [26], and Chlorophyll [27] showed significant results against *S. mutans*. Beta vulgaris [28], 5-aminolevulinic acid (ALA) [29], and *Aloe-emodin* (natural compound isolated from *Aloe vera* and *Rheum palmatum*) [30] showed significant results for *C. albicans* viability reduction. However, the present study showed a higher rate of microbial viability reduction than these previous ones. *E. coli* cultured in a planktonic phase only reached total viability reduction when combining natural compounds with nanocomposites (polydopamine-curcumin nanocomposites), causing photodynamic and photothermal damage [31]. Curcumin-mediated aPDT was also used against *C. acnes*, leading to significant microbial viability results [32], but *C. acnes* was evaluated only in the planktonic phase.

Only one study reports similar results (6 log CFU/mL reduction compared to the negative control) after aPDT with *Myrciaria cauliflora* extracts against *S. aureus*. Thus, the results of the present study are promising for future approaches in aPDT.

None of the plant extracts reduced the microbial viability of *E. coli*, a Gram-negative species often associated with gastro-urinary tract and wound infections and potentially involved in endodontic treatment failures [4,33]. Photosensitizers are known for being effective in inactivating Gram-positive bacteria. However, nearly all photosensitizers reported in the literature demonstrate challenges for inactivating Gram-negative bacteria [25,34,35].

These complications may relate to the composition of the Gram-negative cell wall (phospholipids, lipopolysaccharides, and lipoproteins) and the negative charge of the external cell wall [34]. These characteristics represent a challenge for photosensitizer penetration and therapy effectiveness. Although PDT was effective against *C. albicans*, *E. coli*, and *S. aureus*, single-species biofilms were less susceptible to PDT than the microorganisms in suspension. Our results agree with other findings in the literature [35,36,37].

Biofilm organization provides ecological advantages and increases the resistance against antimicrobials [38]. Furthermore, the extracellular matrix can exclude or limit the access of drugs to microorganisms in deep biofilm layers [39]. Moreover, it is worth noting that biofilms showed significant results with crude plant extracts. Future studies are required to identify and purify the most active fractions, increasing the effectiveness of these natural materials as photosensitizers in PDT.

In order to overcome these challenges confers by Gram-negative cell wall and the extracellular matrix, organic and inorganic nanoparticles have been widely used [40,41,42,43,44]. In a previous systematic review and meta-analysis, the use of inorganic nanoparticles was employed as a drug delivery system facilitating the PS uptake in microbial cells and improving the effect of aPDT [44]. In addition, nanoparticles improve the bioavailable, solubility, selectivity and can be used to overtake the cytotoxicity effect related to possible new drugs and PS [40,41,42,43]. Besides that, antimicrobial peptides can be also utilized in combination with aPDT [45] to overcome these microbial challenge citated above.

Studies suggest that certain microorganisms may have intrinsic chromophores [23,35] and absorb the applied light, potentially causing a more potent response to aPDT. Our experiments analyzed inoculums of *C. albicans*, *C. acnes*, *E. coli*, *S. aureus*, and *S. mutans* in the presence or absence of irradiation and without adding any substance other than nutrient broth. The inoculum used as the control did not show reductions in the number of microorganisms due to the exposure to the light source alone. That suggests that the tested microorganisms do not have natural chromophores or, if they do, the wavelength used in our study does not correspond to that of the intrinsic photoreceptor.

*Senna* spp. extracts produced a high amount of the _1_OH radical, indicating that the main action mechanism of these extracts relates to type I reaction. The _1_OH radical is the most harmful and deleterious reactive oxygen species because it can cause oxidative damage to various cell components, including carbohydrates, lipids, proteins, and nucleic acids, possibly causing cell death. Due to their very short half-life, organisms exposed to this radical cannot develop ways to inhibit its action [46,47]. This outcome has also occurred in commercial photosensitizers, such as methylene blue [48].

Fully understanding the work of *Senna* spp. as a photosensitizer requires identifying and isolating the active photosensitizing agent(s) in plant extracts. As extracts are complex mixtures of compounds, it is also reasonable to consider a synergistic activity of the extract components, showing antimicrobial activity due to two or more compounds acting synergistically. Thus, chromatographic (HPLC) and spectroscopic analyses were performed to investigate the chemical composition of crude extracts of *Senna* spp.

Anthraquinones and flavonoids were the main constituents of the crude extracts of *Senna* spp. identified in the phytochemical study. Fatty acids, steroids, coumarins, and triterpenoids were also identified.

A recent systematic review [10] classified many potential substances for photosensitizer synthesis, including furanocoumarins, polyacetylenes, thiophenes, curcumin, xanthonoids, alkaloids, anthraquinones, phenalenones, porphyrins, and chlorines. The chemical constitution of *Senna* spp. includes anthraquinones [49]. This class of molecules absorbs light in the 300–450 nm wavelength regions and is involved in the enzymatic photodynamic generation of ROS [50].

To the best of our knowledge, there is no study evaluating photosensitizing agents in *Senna* spp. extracts. Thus, further studies are required to verify which of the identified components are photoactive and contribute to the photosensitizing effect of crude extracts.

In cytotoxicity tests on human oral keratinocyte cells cultivated in a monolayer, only *S. alata* did not show cytotoxicity [51]. However, these results should be interpreted carefully because monolayer studies are poor models for predicting human outcomes [52,53]. Additionally, studies have shown that 0.12% chlorhexidine or 1% sodium hypochlorite solution, commonly used in dentistry, presented higher cytotoxicity than *Senna* spp. extracts [36].

Developing new photosensitizers is relevant, mainly to increase the number of therapeutic resources available for treating infectious diseases. In this context, the studied plant materials have a high potential for aPDT use because they are popular plant species in the world, easily acquired, and provide an easy and inexpensive preparation of extracts.

## 5. Conclusions

According to the methodology of this in vitro study, aPDT mediated by plant extracts from *S. macranthera*, *S. splendida*, and *S. alata* is an effective alternative to eliminate *C. acnes*, *C albicans*, *S. aureus*, and *S. mutans*, mainly the *S. alata* plant extract, which presents excellent anti-microbiological results and satisfactory cytotoxicity. This study is the first to report the successful use of these plant materials as photosensitizers.

## Figures and Tables

**Table 1:** Experimental parameters of aPDT using microbial suspensions and biofilm.

Suspension					
Experimental Group	Plant Material Concentrations	Light Dose	Intensity	Pre-Irradiation Time	Irradiation Time in Fractional Mode
Negative control	0	0	0	0	0
Light	0	80 J/cm^2^	155 mW/cm^2^	0	0
Plant material	0.05 mg/mL	0	0	5 m	18 m
PDT	0.05 mg/mL	80 J/cm^2^	155 mW/cm^2^	5 m	18 m
Vehicle control without light exposure	N/A	0	0	0	0
Vehicle control with light exposure	N/A	80 J/cm^2^	155 mW/cm^2^	5 m	18 m
**Biofilm**					
Negative control	0	0	0	0	0
Light	0	139 J/cm^2^	155 mW/cm^2^	0	30 m
Plant material	0.05 mg/mL; 0.50 mg/mL	0	0	15 m	30 m
PDT	0.05 mg/mL; 0.50 mg/mL	139 J/cm^2^	155 mW/cm^2^	15 m	30 m
Vehicle control without light exposure	N/A	0	0	0	0
Vehicle control with light exposure	N/A	139 J/cm^2^	155 mW/cm^2^	15 m	30 m

m = minutes. N/A: not applicable.

**Table 2:** High-resolution MS data from the photoactive compounds.

#	Rt (min)	MF	MM_cal_	[M+H]^+^_cal_	[M+H]^+^_Exp_	Error	[M+Na]^+^_cal_	[M+Na]^+^_Exp_	Error	[M-H]^−^_cal_	[M-H]^−^_Exp_	Error	[Ag+H]^+^_cal_	[Ag+H]^+^_Exp_	Error	[Ag-H]^−^_cal_	[Ag-H]^−^_Exp_	Error
1	22.1	C_27_H_32_O_15_	596.1744	597.1816	597.1806	1.7	619.1636	619.1625	1.7	595.1671	595.1655	2.7	273.0758	273.0764	−2.0	271.0613	no	
2	22.2	C_26_H_34_O_14_	570.1951	571.2024	no		593.1843	593.1827	2.7	569.1878	569.1856	3.9	247.0966	247.0949	6.8	245.0820	no	
3	22.5	C_27_H_30_O_14_	578.1638	579.1711	579.1700	1.8	601.1530	601.1519	1.8	577.1565	577.1541	4.2	255.0653	255.0662	−3.7	253.0507	253.0511	−1.6
4 ^a^	25.1	C_27_H_36_O_15_	584.2108	585.2180	585.2172	1.4	607.2000	607.2015	−2.5	583.2035	583.2029	1.0	261.1122	261.1137	−5.6	259.0977	259.0970	2.6
5 ^a^	25.6	C_28_H_34_O_15_	610.1900	611.1973	611.1957	2.6	633.1792	633.1794	−0.2	609.1827	609.1829	−0.3	287.0915	287.0929	−4.9	285.0769	285.0782	−4.4
6	35.0	C_27_H_32_O_15_	402.0952	403.1025	no		425.0844	no		401.0869	401.0869	2.6	271.0602	no		269.0456	269.0443	4.9
7	35.9	C_15_H_8_O_9_	284.0322	285.0394	no		307.0214	no		283.0249	283.0258	−3.3						
8 ^b^	40.9	C_18_H_14_O_7_	342.0741	343.0813	no		365.0633	no		341.0668	no							
9 ^c^	41.7	nd																

MF = molecular formula; MW = molecular weight; Ag = aglycone; no = not observed; nd = not determined. ^a^ molecular structure not proposed. ^b^ MF proposed based on the relative proportion of [M+Na] + (100%) and [M + 1 *+* Na] + (20%). ^c^ molecular structure and formula not proposed.

**Table 3:** Summary of two-way ANOVA results for bacterial viability in microbial suspensions. The analyzed variables were natural substances (“Natural”) and exposure to light (“Light”).

Source	df	SS	MS	F	*p*-Value	Partial Eta-Squared
***C. albicans***						
Natural	2	41.962	8.392	40,454.530	<0.0001	0.999
Light	4	807.071	201.768	972,589.111	<0.0001	1.000
Natural * Light	8	236.269	11.813	56,944.944	<0.0001	1.000
***C. acnes***						
Natural	2	363.708	90.927	96,920.627	<0.0001	0.999
Light	4	206.363	34.394	36,660.921	<0.0001	0.999
Natural * Light	8	617.759	25.740	27,436.665	<0.0001	1.000
***E. coli***						
Natural	2	83.123	16.625	67,991.599	<0.0001	0.999
Light	4	128.007	32.002	130,881.197	<0.0001	1.000
Natural * Light	8	443.141	22.157	90,618.118	<0.0001	1.000
***S. aureus***						
Natural	2	53.926	10.785	33,985.620	<0.0001	0.999
Light	4	1193.569	298.392	940,281.907	<0.0001	0.999
Natural * Light	8	270.422	13.521	42,607.197	<0.0001	1.000
***S. mutans***						
Natural	2	98.826	19.765	67,209.937	<0.0001	0.999
Light	4	458.872	114.718	390,090.696	<0.0001	1.000
Natural * Light	8	534.762	26.738	90,921.034	<0.0001	1.000

df = degrees of freedom; SS = sum of squares; MS = mean square; F = MS factor/MS residual; *p* = probability of significance, α = 0.050; * interaction between variable analyses.

**Table 4:** Summary of two-way ANOVA results for bacterial viability in microbial biofilm after PDT with plant extracts (0.05 mg/mL or 0.5 mg/mL). The analyzed variables were natural substances (“Natural”) and exposure to light (“Light”).

	Plant Extract at 0.05 mg/mL					Plant Extract at 0.5 mg/mL				
Source	df	SS	MS	F	*p*-Value	df	SS	MS	F	*p*-Value
***C. albicans***										
Natural	2	0.001411	0.0007054	0.9024	0.4087	2	<0.0001	<0.0001	0.007861	>0.9999
Light	4	0.007407	0.00009259	1.184	0.3154	4	<0.0001	<0.0001	0.01032	>0.9999
Natural * Light	8	1320	329.9	422,019	<0.0001	8	1310	327.5	399777	<0.0001
***C. acnes***										
Natural	2	0.0002585	0.0001293	0.09416	0.9102	2	0.9551	0.4775	266.3	<0.0001
Light	4	0.08292	0.02073	15.10	<0.0001	4	8.881	2.220	1238.0	<0.0001
Natural * Light	8	0.03543	0.004428	3.226	>0.05	8	4.433	0.5541	309.0	<0.0001
***E. coli***										
Natural	2	0.01247	0.003238	3.024	0.0528	2	0.003516	0.001758	1.587	0.2094
Light	4	0.7337	0.1843	171.3	<0.0001	4	0.4894	0.1223	110.4	<0.0001
Natural * Light	8	0.01247	0.001559	1.456	0.1823	8	0.008540	0.001068	0.9637	0.4585
***S. aureus***										
Natural	2	0.01741	0.0005750	0.5820	0.5605	2	0.03358	0.01679	12.03	<0.0001
Light	4	0.1198	0.02996	30.32	<0.0001	4	22.56	5.640	4039.00	<0.0001
Natural * Light	8	0.01741	0.002176	2.203	>0.05	8	0.2952	0.03690	26.43	<0.0001
***S. mutans***										
Natural	2	0.5861	0.2930	327.9	<0.0001	2	3.282	1.641	876.1	<0.0001
Light	4	5.119	1.280	1432.0	<0.0001	4	36.26	9.065	4840.0	<0.0001
Natural * Light	8	2.950	0.3688	412.6	<0.0001	8	17.79	2.223	1187.0	<0.0001

df = degrees of freedom; SS = sum of squares; MS = mean square; F = MS factor/MS residual; *p* = probability of significance, α = 0.050; * interaction between variable analyses.

**Table 5:** Summary of two-way ANOVA results for oral keratinocytes viability non-exposed and exposed to light. The analyzed variables were natural substances (“Natural”) and exposure to light (“Light”).

Source	df	SS	MS	F	*p*-Value	Partial Eta-Squared
*Non-light exposed*						
Treatments * Natural	10	348.5	34.85	0.9697	0.4702	0.999
Treatments	5	197,814	39,563	1101	<0.0001	1.000
Natural	2	597.8	298.9	8.317	0.0003	1.000
*Light exposed*						
Treatments * Natural	10	6559	65.59	1.717	0.0769	0.999
Treatments	5	168,348	33,670	881.2	<0.0001	1.000
Natural	2	641.9	321.0	8.400	0.0003	1.000

df = degrees of freedom; SS = sum of squares; MS = mean square; F = MS factor/MS residual; *p* = probability of significance, α = 0.050; * interaction between variable analyses.

## Data Availability

Not applicable.

## Supplementary Materials

The following supporting information can be downloaded at: https://www.mdpi.com/article/10.3390/pharmaceutics15010181/s1, Figure S1: Absorption spectrum of plant extracts diluted in DMSO; Table S1: General information about plant materials included in the present study; Table S2: Microbial Strains and Growth Conditions; Table S3: Summary of results from two-way ANOVA followed by confidence interval estimation to each microorganism cultured on suspension and studied natural substance; Table S4: Summary of results from two-way ANOVA to each microorganism cultured on biofilm and studied natural substance; Table S5: Summary of two-way ANOVA results for ROS detection and natural products. The variables analyses were: type of fluorescence probe (“Probe”) and natural substances (“Natural”); Table S6: Correlation study about formation of hydroxyl radical in photosensitizers and log-reduction found in microorganism after the PDT application.
