# Supplementary material for: In Vitro Evaluation of Photodynamic Activity of Plant Extracts from Senna Species against Microorganisms of Medical and Dental Interest
Source: Pharmaceutics. 2023 Jan 4;15(1):181. doi: 10.3390/pharmaceutics15010181 (PMC9861726; doi:10.3390/pharmaceutics15010181)
Supplement: Supplementary file 1 [file pharmaceutics-15-00181-s001.zip › Suppl_Table S1.pdf]

Supplementary Table S1: General information about plant materials included in the present study

Family	Scientific name	Source	Voucher n.	Mass yield	Coordinates
	Senna splendida (Vogel) H.S.Irwin & Barneby	Leaves / NUBBE LPNBIO	60416	6.4%	S(0513974) W(4053621)
**Fabaceae**	*Senna alata* (Willd.) H.S.Irwin & Barneby	Branches / NUBBE LPNBIO	60420	16.3%	S(0403454) W(3901531)
	Senna macranthera (Collad.) H.S.Irwin & Barneby	Leaves / NUBBE LPNBIO	60422	9.8%	S(0258347) W(3953439)

NUBBE: Núcleo de Bioensaios, Biosíntese e Ecofisiologia de Produtos naturais, UNESP - Institute of Chemistry, Araraquara. LPNBIO: Laboratório de Produtos Naturais e Química Medicinal, UFC- Federal University of Ceará, Fortaleza, Ceará, Brazil. S: South; W: West.
