# Supplementary material for: In Vitro Evaluation of Photodynamic Activity of Plant Extracts from Senna Species against Microorganisms of Medical and Dental Interest
Source: Pharmaceutics. 2023 Jan 4;15(1):181. doi: 10.3390/pharmaceutics15010181 (PMC9861726; doi:10.3390/pharmaceutics15010181)
Supplement: Supplementary file 1 [file pharmaceutics-15-00181-s001.zip › Suppl_Table S2.pdf]

Supplementary Table S2. Microbial Strains and Growth Conditions.

Strain	Culture medium (liquid / solid)	Incubation conditions	Inoculum
C. acnes	TSB enriched with hemin (5 µg/mL)/ RCM	Anaerobiosis at 37 ºC/ 24 h	10^8 CFU/mL
C. albicans	TSB / BHI	Aerobiosis at 37 ºC/ 48 h	10^6 CFU/mL
E. coli	TSB / TSA	Aerobiosis at 37 ºC/ 24 h	10^7 CFU/mL
S. mutans	BHI /BHI	Anaerobiosis at 37 ºC/ 48 h	10^7 CFU/mL
S. aureus	TSB / BHI	Aerobiosis at 37 ºC/ 24 h	10^8 CFU/mL

RCM: *Reinforced Clostridium Medium.* TSB: soy tryptone broth. TSA: soy tryptone agar. BHI: brain and heart infusion.
