# Supplementary material for: In Vitro Evaluation of Photodynamic Activity of Plant Extracts from Senna Species against Microorganisms of Medical and Dental Interest
Source: Pharmaceutics. 2023 Jan 4;15(1):181. doi: 10.3390/pharmaceutics15010181 (PMC9861726; doi:10.3390/pharmaceutics15010181)
Supplement: Supplementary file 1 [file pharmaceutics-15-00181-s001.zip › Suppl_Table S3.pdf]

Supplementary Table S3. Summary of results from two-way ANOVA followed by confidence interval estimation to each microorganism cultured on suspension and studied natural substance.

Characteristics	Study groups Mean ± Confidence Interval (95%) (log CFU / mL)					
	**Control**	**PS-Light**	**PS+Light**	**VC-Light**	**VC+Light**	**P**
C. albicans						
Senna macrantera	5.932±0.023	5.899±0.018	0.000±0.018	5.891±0.025	5.884±0.025	
Senna splendida	5.932±0.023	5.903±0.018	0.000±0.018	5.891±0.025	5.884±0.025	<0.001
Senna reticulata	5.931±0.025	5.906±0.017	0.000±0.018	5.891±0.025	5.884±0.025	
C. acnes						
Senna macrantera	8.100±0.040	7.941±0.040	6.783±0.040	7.932±0.040	7.921±0.040	
Senna splendida	7.988±0.040	7.767±0.038	0.000±0.038	7.932±0.038	7.903±0.038	<0.001
Senna reticulata	7.988±0.040	6.746±0.038	0.000±0.038	7.932±0.038	7.903±0.038	
E. coli						
Senna macrantera	8.908±0.025	8.828±0.020	8.825±0.020	8.856±0.028	8.902±0.028	
Senna splendida	8.908±0.025	8.851±0.020	8.836±0.020	8.856±0.028	8.902±0.028	<0.001
Senna reticulata	8.908±0.025	8.839±0.020	8.810±0.020	8.856±0.028	8.902±0.028	
S. aureus						
Senna macrantera	6.922±0.029	6.870±0.022	0.000±0.022	6.952±0.031	6.914±0.031	
Senna splendida	6.922±0.029	7.569±0.022	0.000±0.022	6.952±0.031	6.914±0.031	<0.001
Senna reticulata	6.922±0.029	7.708±0.022	4.675±0.022	6.952±0.031	6.914±0.031	

Legend. PS-Light: photosensitizer without light; PS+Light: photosensitizer with light; VC-Light: vehicle control without light; VC+Light: vehicle control with light; p<0.05 means significant statistical difference

Supplementary Table S3. Continue. Summary of results from ANOVA TWO-WAY followed by confidence interval estimation to each microorganism and studied natural substance.

	Study groups<br>Mean ± Confidence Interval (95%) (log CFU / mL)					
**Characteristics**	**Control**	**PS-Light**	**PS+Light**	**VC-Light**	**VC+Light**	**P**
S.mutans						
Senna macrantera	8.027±0.021	7.872±0.021	4.834±0.021	7.987±0.030	7.989±0.030	
Senna splendida	8.028±0.023	7.900±0.021	0.000±0.021	7.987±0.030	7.989±0.030	<0.001
Senna reticulata	8.028±0.023	7.926±0.021	0.000±0.021	7.987±0.030	7.989±0.030	

Legend. PS-Light: photosensitizer without light; PS+Light: photosensitizer with light; VC-Light: vehicle control without light; VC+Light: vehicle control with light; p<0.05 means significant statistical difference.
