# Supplementary material for: In Vitro Evaluation of Photodynamic Activity of Plant Extracts from Senna Species against Microorganisms of Medical and Dental Interest
Source: Pharmaceutics. 2023 Jan 4;15(1):181. doi: 10.3390/pharmaceutics15010181 (PMC9861726; doi:10.3390/pharmaceutics15010181)
Supplement: Supplementary file 1 [file pharmaceutics-15-00181-s001.zip › Suppl_Table S4.pdf]

Supplementary Table S4. Summary of results from two-way ANOVA to each microorganism cultured on biofilm and studied natural substance.

	Study groups											
	**Log-reduction of biofilm after PDT with plant extracts (0.05 mg/mL) mean ± standard deviation (log CFU / mL)**						**Log-reduction of biofilm after PDT with plant extracts (0.5 mg/mL) mean ± standard deviation (log CFU / mL)**					
**Characteristics**	**Control**	**PS-Light**	**PS+Light**	**VC-Light**	**VC+Light**	**p-value**	**Control**	**PS-Light**	**PS+Light**	**VC-Light**	**VC+Light**	**p-value**
C. albicans												
S. macranthera	0.04 ± 0.02	0.00 ± 0.02	7.70 ± 0.02	0.05 ± 0.03	0.07 ± 0.04		0.08 ± 0.02	0.01 ± 0.03	7.69 ± 0.03	0.06 ± 0.04	0.08 ± 0.02	
S. splendida	0.04 ± 0.03	0.02 ±0.02	7.70 ± 0.02	0.05 ± 0.03	0.07 ± 0.04	<0.0001	0.08 ± 0.02	0.02 ± 0.03	7.69 ± 0.03	0.07 ± 0.04	0.08 ± 0.02	<0.0001
S. reticulata	0.04 ± 0.02	0.02 ±0.02	7.70 ± 0.02	0.05 ± 0.03	0.07 ± 0.04		0.08 ± 0.02	0.02 ± 0.03	7.69 ± 0.03	0.06 ± 0.04	0.08 ± 0.02	
C. acnes												
S. macranthera	0.02 ± 0.03	0.04 ± 0.04	0.05 ± 0.04	0.06 ±0.03	0.04 ± 0.02		0.06 ± 0.04	0.09 ± 0.03	0.11 ± 0.03	0.22 ± 0.03	0.21 ± 0.04	
S. splendida	0.02 ± 0.03	0.12 ± 0.03	0.00 ± 0.05	0.06 ±0.03	0.04 ± 0.02	0.0026	0.06 ± 0.04	0.14 ± 0.05	1.10 ± 0.05	0.22 ± 0.03	0.21 ± 0.04	<0.0001
S. reticulata	0.02 ± 0.03	0.09 ± 0.03	0.03 ± 0.05	0.06 ±0.03	0.04 ± 0.02		0.06 ± 0.04	0.14 ± 0.07	1.03 ± 0.04	0.22 ± 0.03	0.21 ± 0.04	
E.coli												
S. macranthera	0.02 ± 0.02	0.01 ± 0.03	0.02 ± 0.03	0.20 ± 0.01	0.23 ± 0.04		0.05 ± 0.03	0.01 ± 0.03	0.01 ± 0.05	0.18 ± 0.03	0.18 ± 0.02	
S. splendida	0.02 ± 0.02	0.05 ± 0.05	0.08 ± 0.03	0.20 ± 0.01	0.23 ± 0.04	<0.0001	0.05 ± 0.03	0.03 ± 0.04	0.05 ± 0.04	0.18 ± 0.03	0.18 ± 0.02	<0.0001
S. reticulata	0.02 ± 0.02	0.03 ± 0.04	0.06 ± 0.05	0.20 ± 0.01	0.23 ± 0.04		0.05 ± 0.03	0.04 ± 0.03	0.02 ± 0.04	0.18 ± 0.03	0.18 ± 0.02	
S. aureus												
S. macranthera	0.10 ± 0.04	0.02 ± 0.02	0.05 ± 0.02	0.01 ± 0.02	0.04 ± 0.02		0.17 ± 0.04	0.02 ± 0.04	1.08 ± 0.05	0.01 ± 0.04	0.03 ± 0.03	
S. splendida	0.10 ± 0.04	0.02 ± 0.03	0.03 ± 0.03	0.01 ± 0.02	0.04 ± 0.02	<0.0001	0.17 ± 0.04	0.04 ± 0.02	1.20 ± 0.03	0.01 ± 0.04	0.03 ± 0.03	<0.0001
S. reticulata	0.10 ± 0.04	0.07 ± 0.03	0.02 ± 0.03	0.01 ± 0.02	0.04 ± 0.02		0.17 ± 0.04	0.08 ± 0.04	0.94 ± 0.03	0.01 ± 0.04	0.03 ± 0.03	
S. mutans												
S. macranthera	0.05 ± 0.02	0.01 ± 0.04	0.01 ± 0.03	0.07 ± 0.03	0.07 ± 0.03		0.05 ± 0.04	0.06 ± 0.05	0.09 ± 0.04	0.08 ± 0.04	0.07 ± 0.03	
S. splendida	0.05 ± 0.02	0.01 ± 0.04	0.78 ± 0.04	0.07 ± 0.03	0.07 ± 0.03	<0.0001	0.05 ± 0.04	0.05 ± 0.06	2.08 ± 0.03	0.08 ± 0.04	0.07 ± 0.03	<0.0001
S. reticulata	0.05 ± 0.02	0.02 ± 0.02	0.76 ± 0.02	0.07 ± 0.03	0.07 ± 0.03		0.05 ± 0.04	0.03 ± 0.04	1.82 ± 0.04	0.08 ± 0.04	0.07 ± 0.03	

PS-Light: photosensitizer without light; PS+Light: photosensitizer with light; VC-Light: vehicle control without light; VC+Light: vehicle control with light; p<0.05 means significant statistical difference
