# Supplementary material for: In Vitro Evaluation of Photodynamic Activity of Plant Extracts from Senna Species against Microorganisms of Medical and Dental Interest
Source: Pharmaceutics. 2023 Jan 4;15(1):181. doi: 10.3390/pharmaceutics15010181 (PMC9861726; doi:10.3390/pharmaceutics15010181)
Supplement: Supplementary file 1 [file pharmaceutics-15-00181-s001.zip › Suppl_Table S5.pdf]

Supplementary Table S5. Summary of two-way ANOVA results for ROS detection and natural products. The variables analyses were: type of fluorescence probe (“Probe”) and natural substances (“Natural”)

Source	Df	SS	MS	F	p	Partial Eta-Square
Probe	1	443454379201.389	443454379201..389	228.893	<0.001	0.792
Natural	2	126125468739.611	25225093747.922	13.020	<0.001	0.520
Probe*Natural	2	32430327625.778	6486065525.156	3.348	0.010	0.218

df= degrees of freedom; SS= Sum of squares; MS= mean square; F= MS factor/ MS residual; p= probability of significance, α= 0.050; *interaction between variables analyses
