# Supplementary material for: In Vitro Evaluation of Photodynamic Activity of Plant Extracts from Senna Species against Microorganisms of Medical and Dental Interest
Source: Pharmaceutics. 2023 Jan 4;15(1):181. doi: 10.3390/pharmaceutics15010181 (PMC9861726; doi:10.3390/pharmaceutics15010181)
Supplement: Supplementary file 1 [file pharmaceutics-15-00181-s001.zip › Suppl_Table S6.pdf]

Supplementary Table S6. Correlation study about formation of hydroxyl radical in photosensitizers and log-reduction found in microorganism after the PDT application.

Microorganism	Photosensitizer	*p*-value	CI (95%)	r (Pearson)	R^2	Power (95%)
C. acnes						
	S. splendida	0.3811	-0.58; 0.92	0.4412	0.1947	0.2045
	S. alata	0.0359	0.09; 0.98	0.8409	0.7071	0.6828
C. albicans						
	S. macrantera	0.0578	-0.04; 0.98	0.7966	0.6345	0.5457
	S. splendida	0.0230	0.21; 0.99	0.8733	0.7627	0.7543
	S. alata	0.0638	-0.07; 0.98	0.7859	0.6177	0.5762
S. aureus						
	S. macrantera	0.0020	0.69; 1.00	0.9628	0.9271	0.9633
	S. splendida	0.0003	0.88; 1.00	0.9863	0.9727	0.9961
	S. alata	0.0020	0.70; 1.00	0.9636	0.9285	0.9647
S. mutans						
	S. macrantera	0.4230	-0.60; 0.92	0.4071	0.1657	0.1844
	S. splendida	0.2415	-0.45; 0.94	0.5661	0.3204	0.2968
	S. alata	0.3661	-0.57; 0.92	0.4537	0.2058	0.2122

CI: confidence interval; r: correlation coefficient; R^2: determination coefficient. P-value <0.05 was considered as statistical significance
