# Supplementary figures and images for: In Vitro Evaluation of Photodynamic Activity of Plant Extracts from Senna Species against Microorganisms of Medical and Dental Interest
Source: Pharmaceutics. 2023 Jan 4;15(1):181. doi: 10.3390/pharmaceutics15010181 (PMC9861726; doi:10.3390/pharmaceutics15010181)

**Supplementary Figure S1.** Absorption spectrum of plant extracts diluted in DMSO.

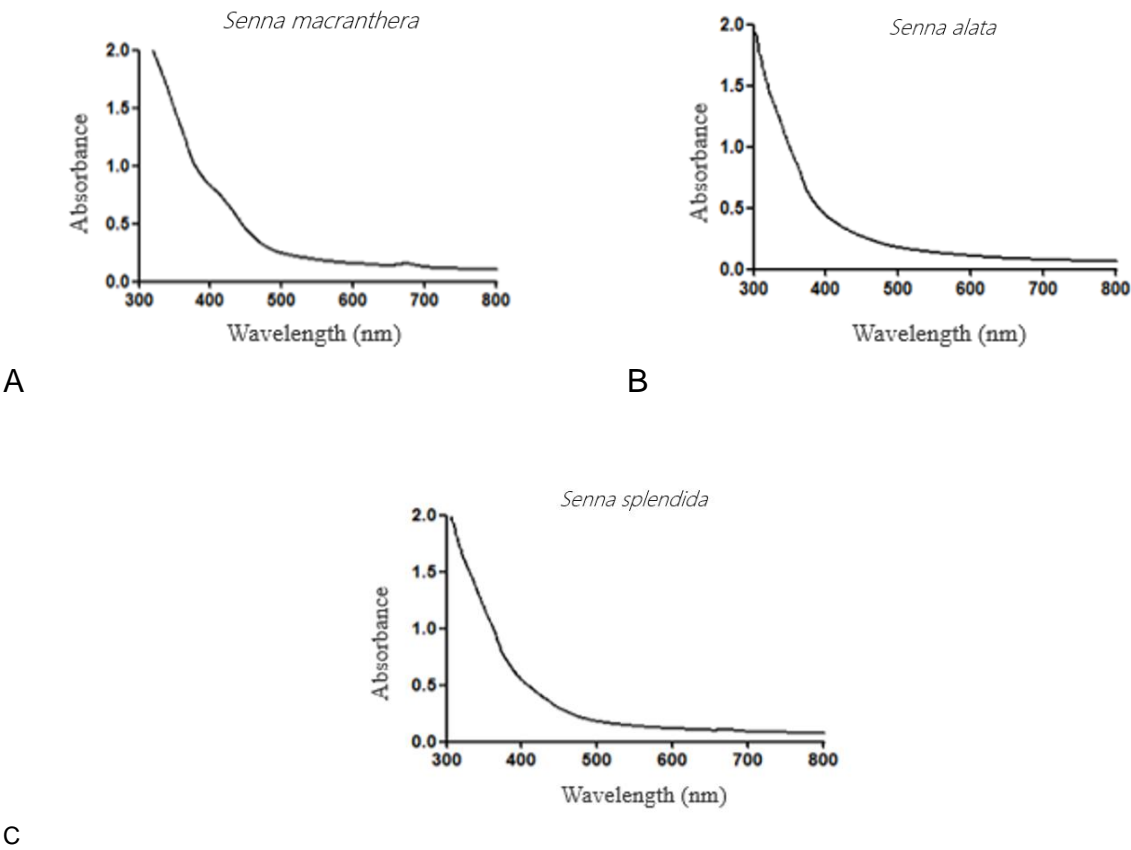

Supplement: Supplementary file 1 [file pharmaceutics-15-00181-s001.zip › Supplementary Figure S1.pdf]
